# Long-term health after Severe Acute Malnutrition in children and adults- the role of the Pancreas (SAMPA): Protocol

**DOI:** 10.12688/f1000research.123389.2

**Published:** 2022-10-03

**Authors:** Sana Ahmed, George PrayGod, Nanette R. Lee, Paul Kelly, Geeta Trilok-Kumar, Molly Chisenga, Belinda Kweka, Daniel Faurholt-Jepsen, Rikke Krogh-Madsen, James AM Shaw, Dixi M. Paglinawan-Modoc, Juan Solon, Mette Frahm Olsen, Darko Stefanovski, Sharon Cox, Dorothea Nitsch, Ruth Keogh, Suzanne Filteau

**Affiliations:** 1 Institute of Home Economics, University of Delhi, Delhi, 110016, India; 2 National Institute for Medical Research, Mwanza, Tanzania; 3 USC-Office of Population Studies Foundation, Inc., University of San Carlos, Populations Studies Foundation, Cebu City, 6000, Philippines; 4 University Teaching Hospital, Lusaka, Zambia; 5 Faculty of Medicine and Dentistry, Queen Mary University of London, London, UK; 6 Delhi School of Public Health, Institution of Eminence, University of Delhi, Delhi, 110007, India; 7 Department of Infectious Diseases, Rigshospitalet, Copenhagen, 2100, Denmark; 8 Centre for Physical Activity Research, Copenhagen University Hospital, Rigshospitalet, Copenhagen, Denmark; 9 Department of Infectious Diseases, Copenhagen University Hospital, Amager and Hvidovre, Hvidovre, Denmark; 10 Translational and Clinical Research Institute, Newcastle University, The Medical School, Framlington Place, Newcastle-upon-Tyne, UK; 11 Nutrition Center of the Philippines, Muntinlupa City, Manila, Philippines; 12 Department of Nutrition, Exercise and Sports, University of Copenhagen, Copenhagen, 1017, Denmark; 13 Department of Clinical Studies, New Bolton Center, University of Pennsylvania School of Veterinary Medicine, Philadelphia, USA; 14 Faculty of Epidemiology and Population Health, London School of Hygiene and Tropical Medicine, London, WC1E 7HT, UK

**Keywords:** Malnutrition, wasting, diabetes, pancreas, non-communicable diseases, oral glucose tolerance test

## Abstract

**Background**: Prenatal growth retardation may increase the risk of later chronic non-communicable diseases (NCDs), including diabetes; however, long-term effects of wasting malnutrition in childhood or adulthood are less studied. Pancreatic exocrine and endocrine functions, both critical for nutrition and NCD aetiology, may not fully recover following malnutrition. However, the evidence and mechanistic information is piecemeal. We hypothesise that wasting malnutrition at any age has long-term detrimental effects on endocrine and exocrine pancreatic structure and function.

**Methods: **The SAMPA international research programme will assess pancreatic structure and function in 3700 participants from ongoing observational nutrition cohorts, two adolescent and four adult, in Zambia, Tanzania, Philippines, and India. Pancreas size, structure, and calcification will be assessed by ultrasound and computed tomography (CT) scan; exocrine function by faecal elastase and serum lipase; and endocrine function by haemoglobin A1c (HbA1c) and blood glucose, insulin and C-peptide concentrations during an oral glucose tolerance test (OGTT). In-depth hormonal analyses of incretins, glucagon, proinsulin and trypsinogen during OGTT and intravenous glucose tolerance tests will be done in subsets of adult participants. Pancreatic size and function outcomes will be compared between people with and without prior wasting malnutrition. Analyses will investigate effect modification by sex, current age, time since malnutrition, current body mass index and dietary patterns. Mathematical modelling of OGTT data will be used to estimate the relative contribution to glucose dysregulation of decreased insulin production, changes in insulin clearance and increased insulin resistance. Proinsulin/insulin ratio will be analysed in archived samples from the Tanzanian cohort using a nested case-control design to investigate whether abnormal values precede diabetes.

**Conclusions:** SAMPA, a large-scale multi-centre research programme using data from people with or without prior wasting malnutrition to assess several aspects of pancreatic phenotype, will provide coherent evidence for future policies and programmes for malnutrition and diabetes.

## Introduction

There is an increasing prevalence of overweight and obesity worldwide which is associated with increases in non-communicable diseases (NCDs), including diabetes. ^1^ Meanwhile, wasting malnutrition remains common in low- and middle-income countries (LMICs). ^2^ It is well-documented that prenatal malnutrition, usually measured as low birth weight, can increase the risk of NCDs in later life, ^3^ but how wasting malnutrition in childhood or adulthood may affect long-term health is less studied. ^4^ The issue of child or adult malnutrition's long-term implications is becoming increasingly important because more children survive severe acute malnutrition as a result of improved care, while pharmacological treatments for infections, such as HIV and tuberculosis, that commonly cause or are linked with malnutrition, have increased patient survival among all age groups. Evidence from a recent systematic review suggests pancreatic endocrine and exocrine functions may not recover fully after childhood or adult wasting malnutrition. ^5^ Such long-term effects on the pancreas may contribute to the different phenotype of diabetes common in LMICs with prevalent malnutrition, compared with the diabetes phenotype in high-income countries. Diabetes in LMICs often has a relatively early disease onset (<50 years) and may occur at a low or normal body mass index (BMI). ^6^ There are heterogeneous reports of atypical diabetes in LMICs, describing an entity of malnutrition-related, insulin-deficient diabetes related to pancreatic damage, for example, a phenotype termed fibrocalculous pancreatic diabetes, ^7^ but these studies often report highly selected patients, which can provide mechanistic but not epidemiologic information, ^8^ or precede the availability of modern assessment methods. ^9^ Our own work has shown that prior malnutrition in Tanzanian adults is associated with lower insulin production in men ^10^ and that insulin deficiency is a more common cause of diabetes than insulin resistance. ^11^ If malnutrition is associated with an atypical form of diabetes, driven by insulin deficiency and not resistance, then this has major implications for health policy, planning, investigation and treatment of diabetes in LMICs, because current first-line treatments of diabetes in these settings usually use treatments such as metformin which would be inappropriate for insulin-deficient diabetes.

Both pancreatic endocrine ( *i.e.* production of hormones such as insulin) and exocrine ( *i.e.* production of enzymes to aid digestion) functions are critical for nutritional metabolism and in chronic diseases including diabetes. Furthermore, deficits in exocrine and endocrine functions may interact such that diabetes is common among people with exocrine pancreas insufficiency ^12^ and low faecal elastase is common in patients with type 2 diabetes, especially those with poor glycaemic control. ^13^ There is a dearth of studies explaining the mechanisms of the exocrine-endocrine interactions leading to abnormalities in both functions.

In spite of its central importance, there has been surprisingly little research on pancreatic function following recovery from wasting malnutrition. This is likely due, in part, to there being no validated, easily measurable indicator of prior wasting malnutrition, in contrast to studies of poor early linear growth, resulting in largely irreversible stunting, short legs, and increased sitting/standing height ratio in adulthood. There is thus an urgent need for prospective cohort studies to investigate long-term effects of wasting malnutrition.

**Figure 1.**
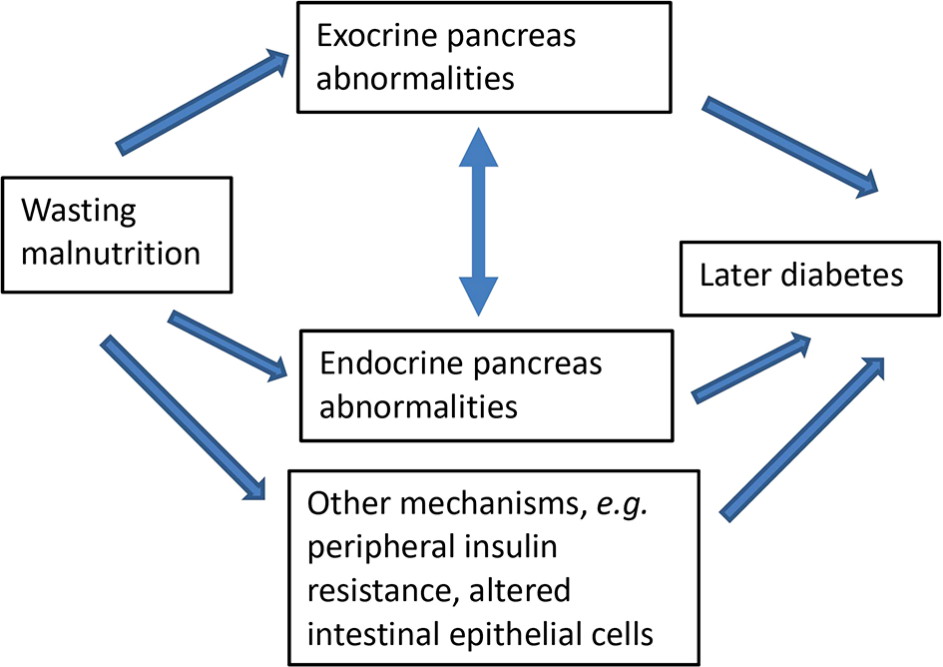
Conceptual framework linking wasting malnutrition with later impaired endocrine and exocrine pancreas function.

The central hypothesis of the severe acute malnutrition in children and adults - the role of the pancreas (SAMPA) study is that an episode of wasting malnutrition, irrespective of age of occurrence, affects endocrine and exocrine pancreatic size, structure and function. The study will recall for cross-sectional investigations participants from established cohorts recruited for the research team’s previous studies in Tanzania, Zambia, India and the Philippines. Many cohort members were originally recruited because of an episode of severe malnutrition or their infection with HIV or tuberculosis; their prior nutritional status, plus those of well-nourished controls, is documented. The cohorts now range in age from adolescence to late middle age and live in places with a wide range of access to foods high in fat and sugar which, combined with a more sedentary lifestyle, could affect their risk of diabetes. We will assess the association of prior wasting malnutrition with current pancreas structure and function, taking into account factors such as sex, current age, time since malnutrition, current BMI and body composition, and infections which could affect pancreas functions. Figure 1 shows the conceptual framework guiding the programme of research.

The above relationships may be subject to modification by sex, age at pancreas assessment, time since malnutrition, BMI, diet, or HIV status.

## Protocol

### Study objectives and hypotheses

*Overall hypothesis*

Wasting malnutrition at any age has medium- and long-term detrimental effects on endocrine and exocrine pancreatic function and structure.

1. ***Specific objective 1*:** To investigate whether wasting malnutrition at various ages is associated with abnormalities of pancreas structure and function later in life.
  a. ***Hypothesis 1*:** Prior wasting malnutrition is associated with later abnormal pancreatic structure and endocrine and exocrine function.
  b. ***Pancreatic phenotype of interest:*** Diabetes assessed by oral glucose tolerance test (OGTT) and haemoglobin A1c (HbA1c), exocrine pancreatic function tests (serum lipase, faecal elastase), pancreatic size and architecture measured using ultrasound for all participants and measured using computed tomography (CT) scans for a subset of participants (non-pregnant adult participants).
2. ***Specific objective 2:*** To investigate whether pancreatic abnormalities in participants with prior malnutrition and diabetes are analogous to a previously described entity of fibro-calculous diabetes.
  a. ***Hypothesis 2:*** Prior wasting malnutrition is associated with pancreatic calcification which in turn is associated with diabetes.
  b. ***Pancreatic phenotype of interest:*** Pancreatic calcifications determined from CT scans among non-pregnant adult study participants.
3. ***Specific objective 3:*** To investigate the relative importance of decreased insulin production or increased insulin resistance in malnutrition-associated diabetes.
  a. ***Hypothesis 3:*** The abnormal glucose regulation seen after wasting malnutrition is associated with relative insulin deficiency with or without insulin resistance.
  b. ***Pancreatic phenotype of interest:*** Indices of glucose metabolism and insulin kinetics based on mathematical models of glucose and hormone concentrations during OGTT and intravenous glucose tolerance tests (IVGTT). Homeostasis model assessment of insulin resistance (HOMA-IR) and liver fat (insulin resistance) will also be assessed.
4. ***Specific objective 4:*** To investigate whether a prior abnormal pro-insulin/insulin ratio is associated with diabetes in adults infected or not with HIV or previously with tuberculosis.
  a. ***Hypothesis 4:*** An abnormal pro-insulin/insulin ratio is associated with later development of diabetes in adults who had malnutrition earlier in life in association with HIV or tuberculosis infection.
  b. ***Pancreatic phenotype of interest:*** Fasting proinsulin/insulin ratio among Chronic Infections, Co-morbidities and Diabetes in Africa (CICADA) cohort participants measured several years before the SAMPA investigations.

### Study cohorts

**Figure 2.**
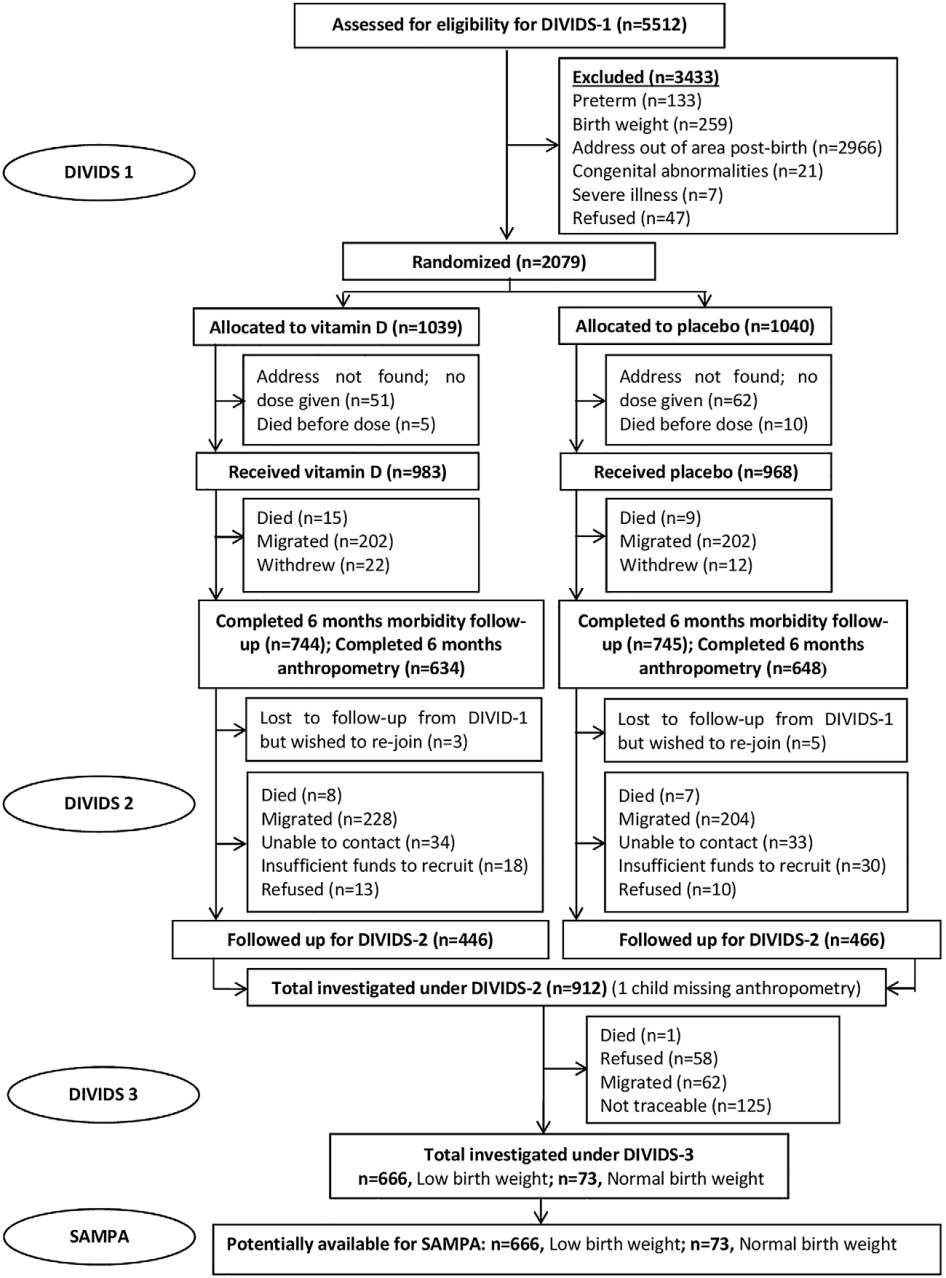
Cohort flow chart for the DIVIDS (Delhi Infant Vitamin D Supplementation) study, India.

**Figure 3.**
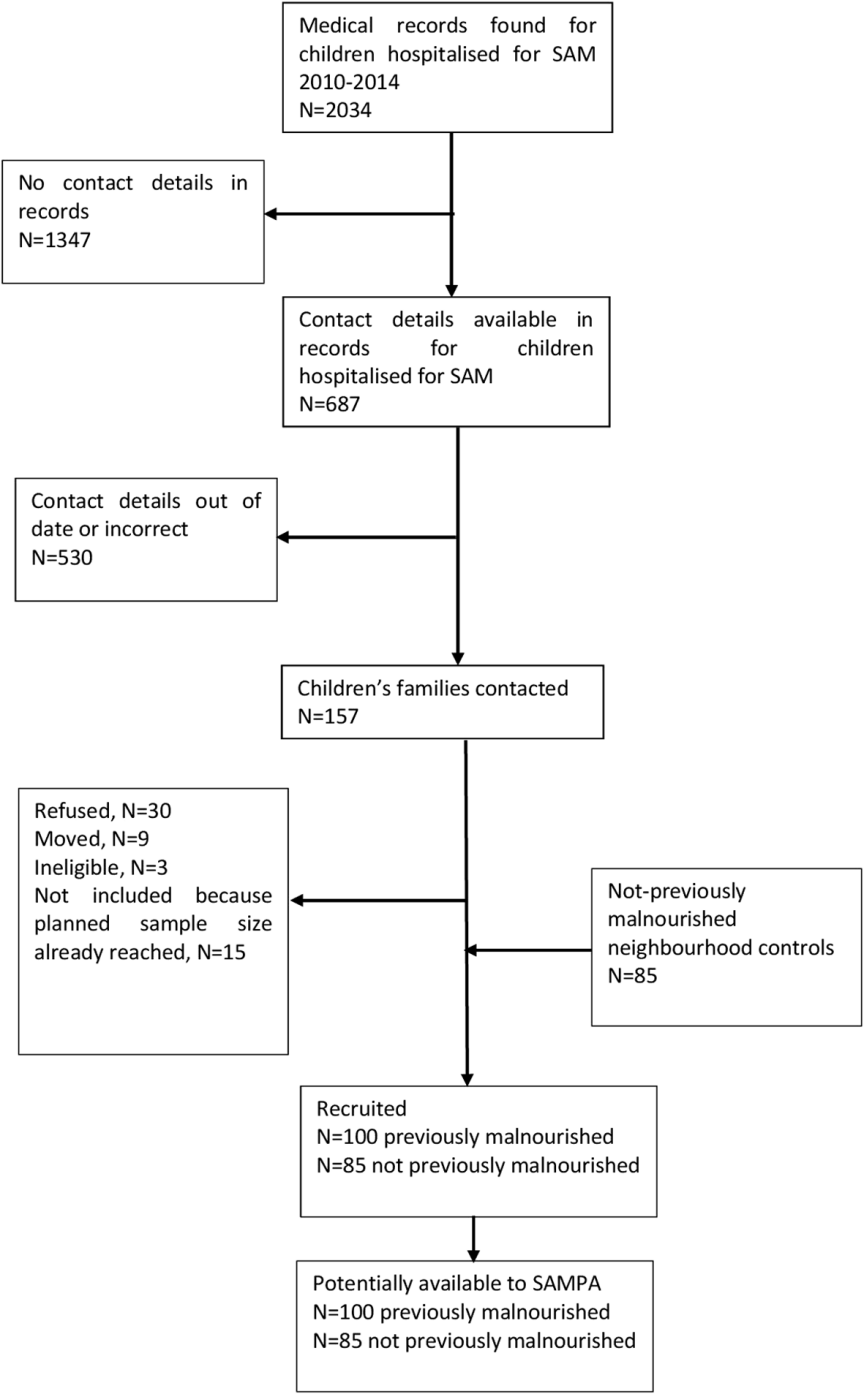
Cohort flow chart for SAM (Severe Acute Malnutrition), Lusaka, Zambia.

**Figure 4.**
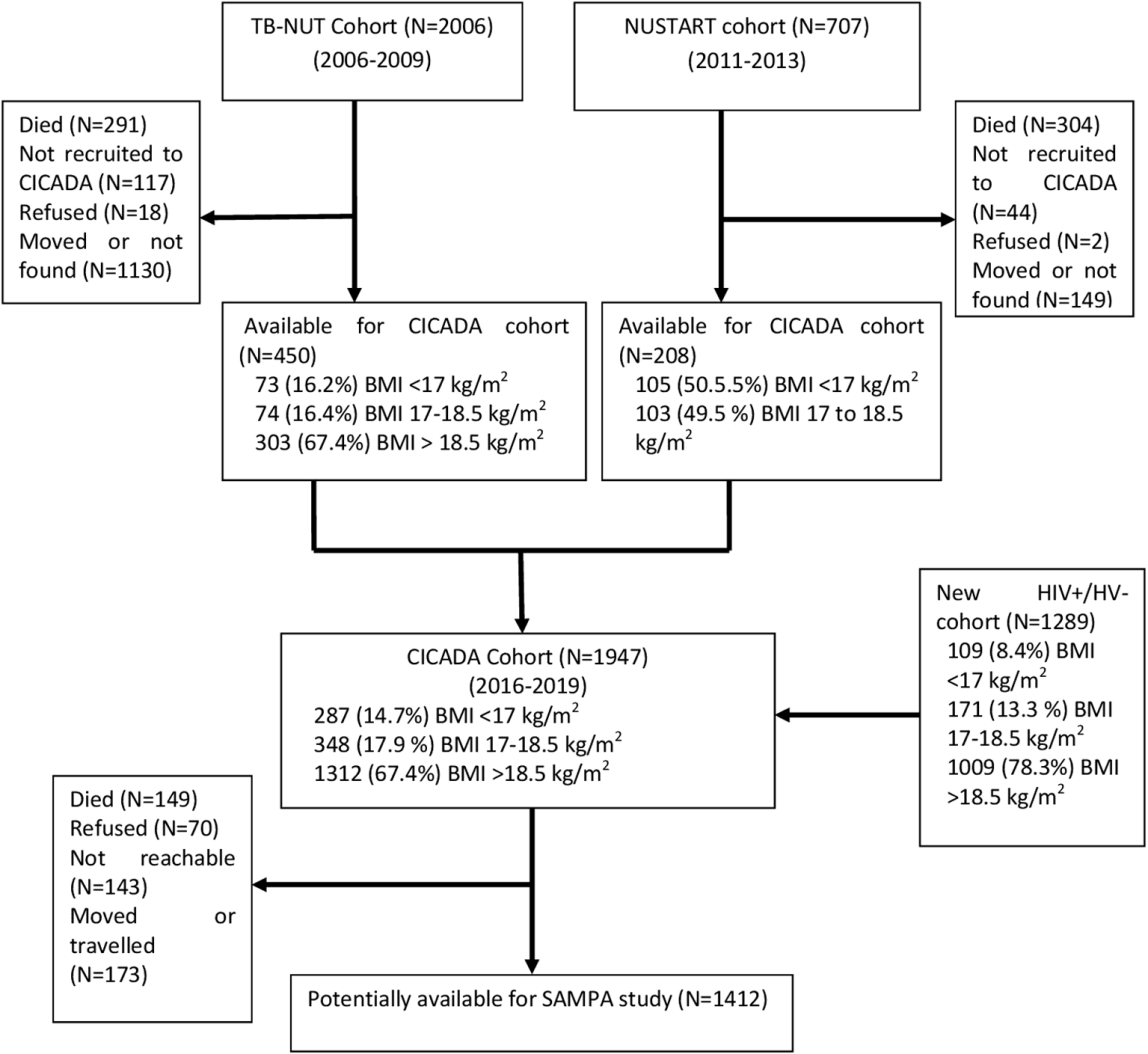
Cohort flow chart for CICADA (Chronic Infections, Co-morbidities and Diabetes in Africa), Mwanza, Tanzania.

**Figure 5.**
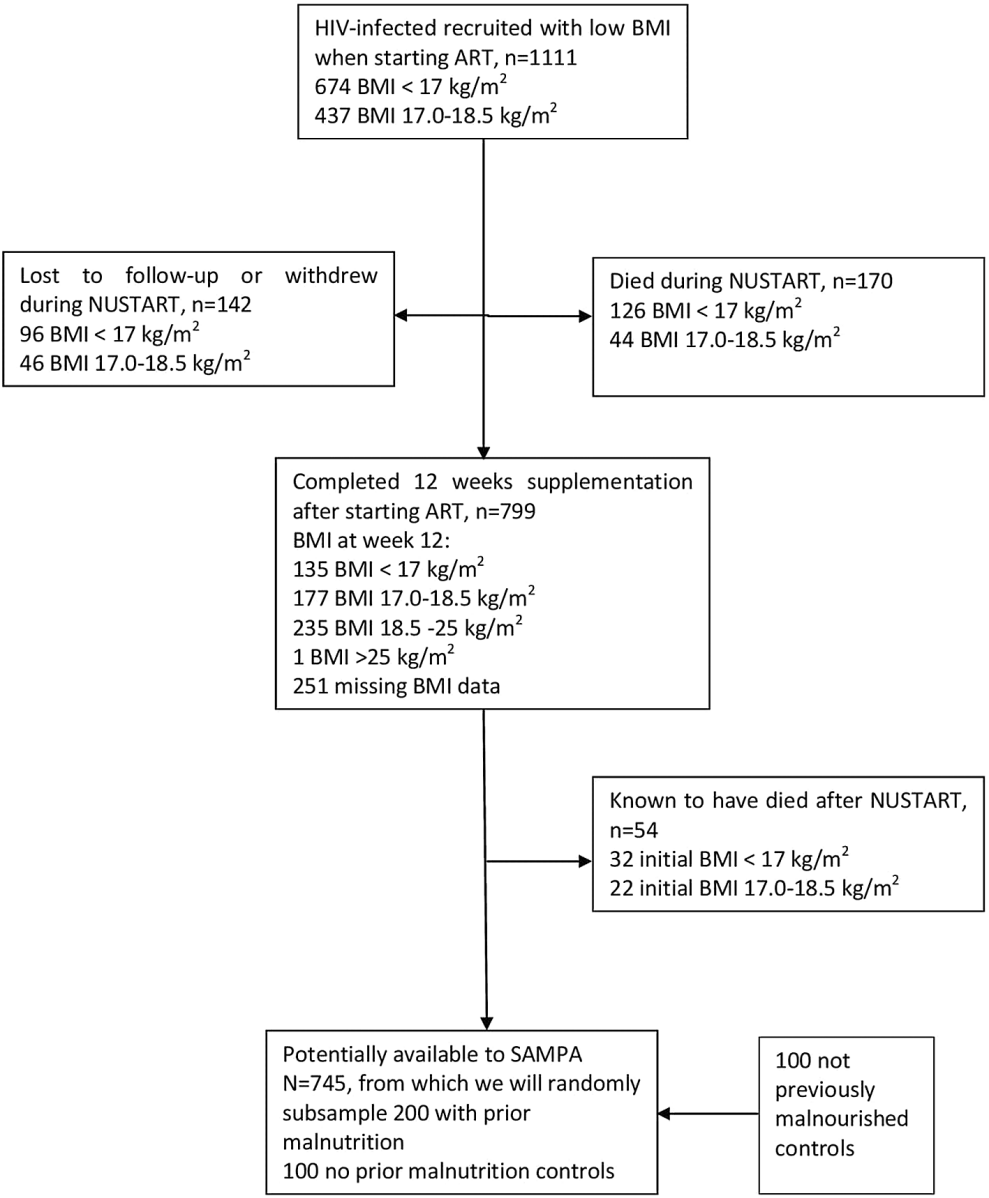
Cohort flow chart for NUSTART (Nutritional Support for Africans Starting Antiretroviral Therapy), Lusaka, Zambia.

**Figure 6.**
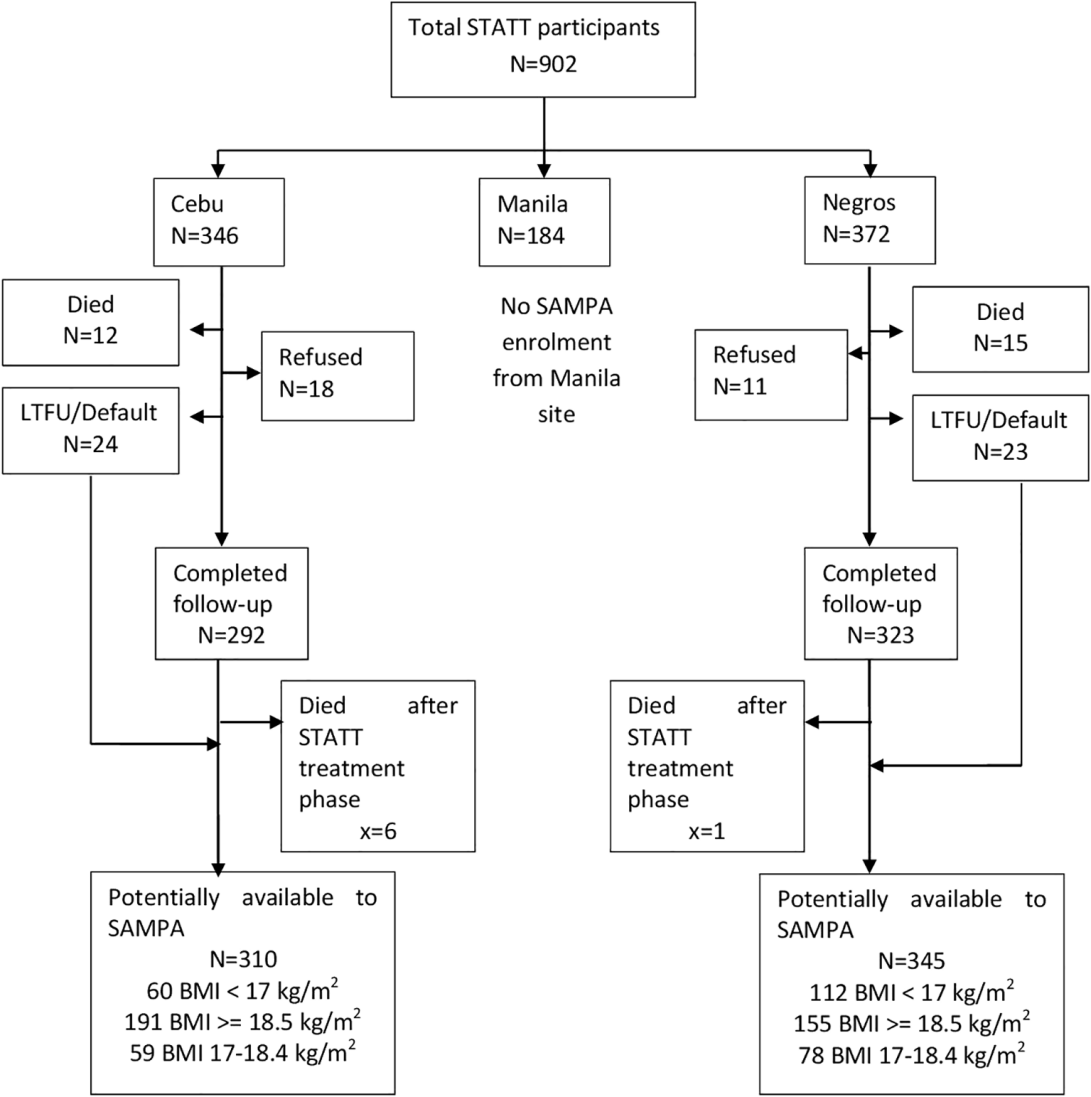
Cohort flow chart for St-ATT (Starting Anti-TB Treatment) Cohort, Philippines.

**Figure 7.**
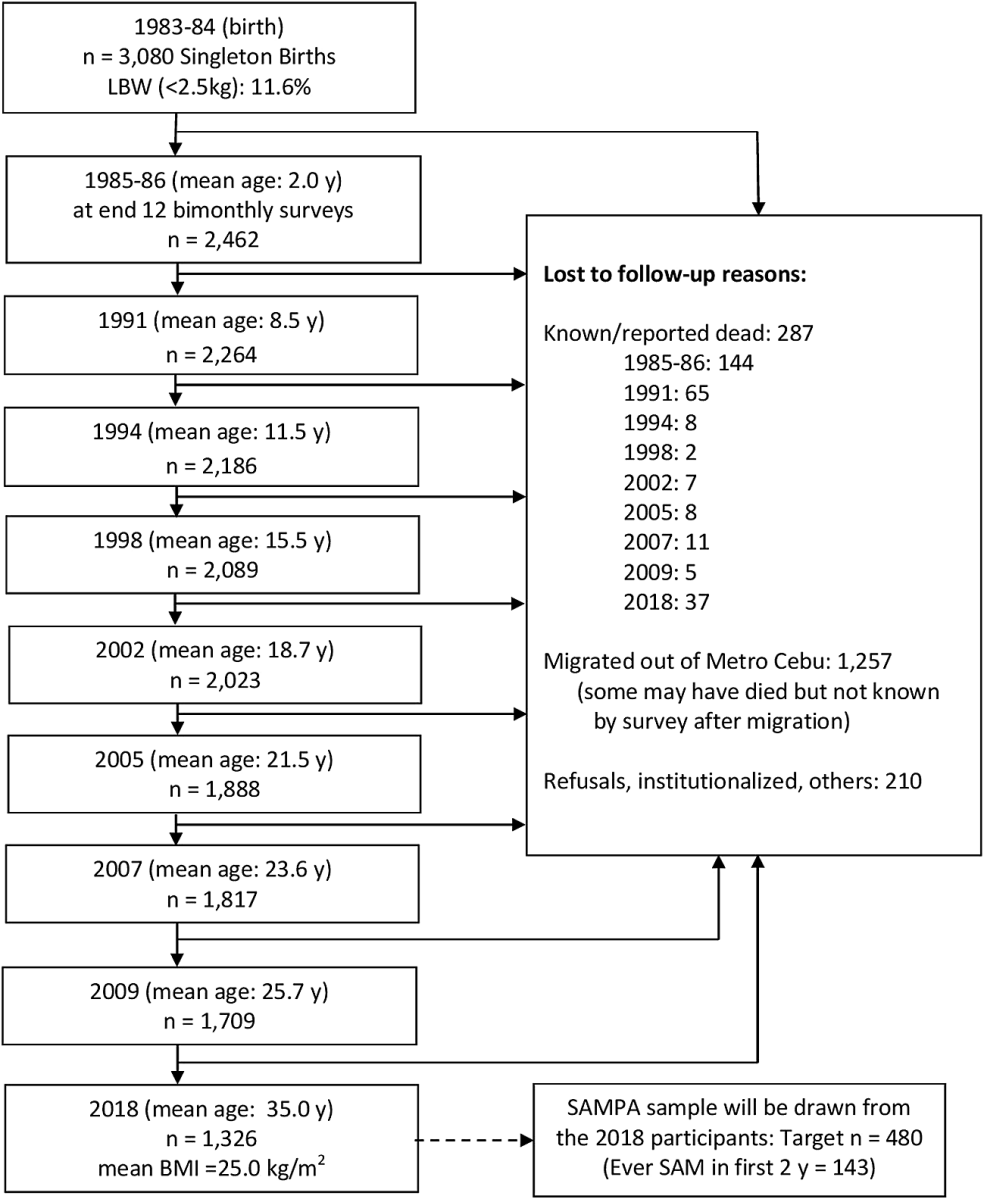
Cohort flow chart for CLHNS (Cebu Longitudinal Health and Nutrition Survey), Philippines.

This study brings together diverse cohorts ( Table 1) under a common theme providing participants with prospectively assessed and clearly defined malnutrition exposure across populations and life-course and not previously malnourished controls, all from LMICs at various stages of the nutrition transition but with ongoing burden of infectious disease. Flow charts for each cohort are given in Figures 2– 7.

**Table 1.** Study cohorts for Severe Acute Malnutrition in children and adults - the role of the Pancreas (SAMPA).

	Cohort	Sample size-previous and expected
**1.**	***DIVIDS (Delhi Infant Vitamin D Supplementation) study, India*** The DIVIDS cohort comprises infants born low birth weight (LBW, <2.5 kg) at term in 2007-2010. They had monthly follow-up until 6 months ^14^ then at 5 years ^15^ and are currently, aged ~13 years. *For SAMPA, the DIVIDS cohort acts as a positive control since we expect adverse long-term non-communicable disease consequences of being born LBW.*	• Birth: 2079 total, all LBW • Age 5 y: 912 total, 764 (84%) BMIZ>-2, 138 (15%) BMIZ -2 to -3, 9 (1%) BMIZ<-3; 1 no anthropometry • Age ~13 y (ongoing): 647 total, 482 (75%) BMIZ>-2, 117 (18%) BMIZ -2 to -3, 48 (7%) BMIZ<-3 • Expected for SAMPA: 750 total
**2.**	***SAM (Severe Acute Malnutrition), Lusaka, Zambia*** This group comprises: a) 100 children who were hospitalised with malnutrition at University Teaching Hospital (UTH) between 2010 and 2014 when they were < 2 years b) 85 never-malnourished neighbourhood controls. Children were aged ~9 years at previous follow-up ^16^ and will be ~12 years for SAMPA.	• Age < 2 y: 100 malnutrition • Age 9 y: 185 total, 100 previous malnutrition, 85 no malnutrition; currently 17 (9%) BMIZ <-2 • Expected for SAMPA: 180 total
**3.**	***CICADA (Chronic Infections, Co-morbidities and Diabetes in Africa), Mwanza, Tanzania*** The CICADA cohort, ^17^ total n=1947, comprises three subgroups: a) Tuberculosis patients and controls from the TB-NUT study whose HIV and nutritional status ~16 years ago at recruitment are known (50% BMI<18.5 kg/m ^2^) as well as more recently recruited HIV-infected and uninfected people (controls) ^18^^,^^19^ b) NUSTART, conducted 2010-2014, was a two-site (Mwanza and Lusaka) trial of food supplements for malnourished (BMI<18.5 kg/m ^2^) HIV-infected adults, recruited when starting antiretroviral therapy. ^20^ In Mwanza, 704 participants were recruited, 409 were alive and followed at the 12-week NUSTART end-point; 273 were available at a follow-up two-three years after starting ART and 206 were recruited to the CICADA study. c) HIV-infected and uninfected people, both malnourished and well-nourished, recruited 3-four years ago for HIV, nutrition and diabetes assessments *CICADA has the most detailed longitudinal diabetes data of the project cohorts, archived fasting samples, and are the oldest so have had longer to develop diabetes; therefore, this cohort will be used for the in-depth and longitudinal components.*	CICADA (TB-NUT) 16 y prior: 450 total • 73 (16.2%) BMI <17 kg/m ^2^ • 74 (16.4%) BMI 17 to 18.5 kg/m ^2^ • 303 (67.4%) BMI > 18.5 kg/m ^2^ CICADA (NUSTART) 11 y prior: 208 total • 105 (50.5%) BMI <17 kg/m ^2^ • 103 (49.5 %) BMI 17 to 18.5 kg/m ^2^ CICADA (New HIV +/- cohort) 4 y prior: 1293 total • 109 (8.4%) BMI <17 kg/m ^2^ • 171 (13.3 %) BMI 17 to 18.5 kg/m ^2^ • 1009 (78.3%) BMI >18.5 kg/m ^2^ Expected for SAMPA: 1412 total
**4.**	***NUSTART, Lusaka, Zambia*** 200 previously malnourished HIV-infected adults will be traced and recruited from the NUSTART Lusaka participants. ^20^ We will also recruit 50 HIV-infected and 50 non-HIV-infected, not previously malnourished, neighbourhood controls.	• 10 y prior: 1111 total, 437 (39%) BMI 17 to 18.5 kg/m ^2^, 674 (61%) BMI<17 kg/m ^2^ • Expected for SAMPA: 300 total
**5.**	***St-ATT (Starting Anti-TB Treatment) Cohort, Philippines*** Between Aug 2018 and Feb 2020, the St-ATT cohort recruited 900 adults undergoing tuberculosis treatment in three provinces: Cebu, Negros Occidental and Metro Manila, encompassing urban, peri-urban and rural populations. 17% had HbA1c >=7.0% (probable diabetes in TB) with an additional 30% with HbA1c 5.8%-7.0% (‘prediabetes’/mild TB-induced hyperglycaemia). ^21^ https://www.isrctn.com/ISRCTN16347615 *This cohort will be involved in the in-depth analyses.*	• 1-2 y prior: 900 total, 495 BMI >18.5 kg/m ^2^, 189 (21%) BMI 17.0-18.5 kg/m ^2^, 216 (24%) BMI <17 kg/m ^2^ • Expected for SAMPA: 600 total
**6.**	***CLHNS (Cebu Longitudinal Health and Nutrition Survey), Philippines*** The mothers of the cohort were randomly selected in 1983-84 when they were pregnant. ^22^ All singleton births were included regardless of nutrition status. Since the original bimonthly follow-up surveys up to age two years, there have been 8 follow-up surveys including in 2018-2019 when 1326, ~43% of the initial birth cohort (3,080), were available. *This cohort represents our longest follow-up of malnutrition diagnosed by anthropometry.* (Earlier cohort data are available open access: https://www.cpc.unc.edu/projects/cebu).	• Birth: 28 LBW of the 143 with childhood malnutrition • Age ≤ 2 y: of 420 total to be included, 143 (34%) WHZ<-3 • Expected for SAMPA: 420 total

### Participant recruitment

Cohort members or their guardians (in case of children) will be contacted by the site principal investigators using the participant contact details from their most recent follow-ups. They will be asked if they wish to participate in SAMPA, and written informed consent will be obtained. For NUSTART, Lusaka, we will need to recruit never-malnourished controls. There are several possible levels of participation in SAMPA, based on cohort, sample size needed for testing specific hypotheses, and individual participants’ willingness to consent to specific tests. The potential different levels of participation are below. People not consenting to either version of the OGTT, a) or b), will be considered as not consenting to SAMPA overall and thus ineligible:

a) Questionnaires for socio-economic demographic and dietary assessment; anthropometry and body composition; medical exam; urine and stool sample collection; abdominal ultrasound and OGTT with blood sampling at baseline (fasting), and 15, 30, 45, 60, 90 and 120 minutes after glucose ingestion;
b) Questionnaires for socio-economic demographic and dietary assessment; anthropometry and body composition; medical exam; urine and stool sample collection; abdominal ultrasound and minimal OGTT only, that is, with blood sampling at baseline (fasting), and 60 and 120 minutes after glucose ingestion;
c) Addition of CT scans to a) or b). Children will not be asked to have CT scans. Women of childbearing age will not be asked to have CT scans unless they consent to a pregnancy test and have a negative result;
d) Addition of IVGTT. This will be requested only for subsets of participants in the CICADA cohorts, as detailed below; glucose infusion will be at 0 minutes and samples will be collected at -10, -1, 2, 4, 6, 8, 10 minutes using an indwelling cannula.

Regarding point c) above, although children in the DIVIDS cohort will not have CT scans, 100 of them will have MRI scans, funded by a separate project, which will be able to assess pancreas size and structure, as well as visceral fat.

### Outcome measures

*Primary outcome measures*

1. Pancreatic endocrine function: diabetes or impaired glucose tolerance (pre-diabetes) by OGTT or HbA1c, defined using World Health Organization (WHO) cut-offs ^23^
2. Pancreatic exocrine function: faecal elastase and serum lipase

*Secondary outcome measures*

1. Pancreas size and structure: Pancreas size and architecture using ultrasound and CT (subsets from all adult cohorts)
2. Pancreatic calcification: Calcification determined by CT scan (subsets from all adult cohorts)
3. Insulin production and insulin resistance:
  a. Mathematical modelling of blood glucose, insulin and C-peptide at three (for children) or seven (for adults) times during a 120-minute OGTT for estimation of indices of first phase, second phase and total insulin release, insulin kinetics and insulin resistance;
  b. Mathematical modelling of data from analytes from IVGTT measured in blood samples collected at -10, -1, 2, 4, 6, 8, 10 minutes: glucose, insulin, and C-peptide (subset from CICADA *).*
4. Other hormonal contribution to glucose metabolism and diabetes: Incretins (gastric inhibitory polypeptide (GIP) and glucagon-like peptide-1 (GLP-1)) and glucagon at 0, 30, 60, 90 and 120 minutes during an OGTT (subsets from CICADA and St-ATT). The goal is to assess whether insulin resistance is associated with changes in endocrine signals from the gut. The area under the curve of glucagon (AUC glucagon) will be used to assess a possible presence of hyperglucagonemia, often seen in T2D subjects.
5. Proinsulin: Proinsulin in baseline samples before OGTT. The baseline proinsulin levels will be used as indicator of β-cell stress/exhaustion in the fasting state where higher levels of proinsulin will indicate higher levels of β-cell stress.
6. Abnormal prior pro-insulin/insulin ratio and diabetes (subset): From the CICADA cohort, measurement of pro-insulin and insulin in fasting samples collected four years previously and comparison with current diabetes by OGTT. Like the fasting proinsulin, the pro-insulin/insulin ratio in fasting samples will be used as a surrogate measure of β-cell stress.

### Assessments

The following assessments will be carried out on all consenting participants across the cohorts. Target sample sizes are as in Table 1.

1. ***OGTT:*** The primary outcome for comparing those with and without prior malnutrition across all cohorts will be glucose ≥ 7.8 mmol/L at 2 hours in a standard OGTT with 75 g glucose (in children, 1.75 g/kg body weight up to 75 g) that is, diabetes plus impaired glucose tolerance by WHO criteria. ^23^ Glucose in one hour samples in all participants will also be measured since this may be a better predictor of progression to diabetes due to insulin deficiency than the two-hour time point. ^24^ Those with OGTT compatible with diabetes will be called back for a repeat OGTT in order to determine need for referral for clinical care. Urine dipstick will also be done for ketones to assess potential need for (short-/long-term) insulin replacement therapy. Indices of beta-cell function, insulin kinetics, and insulin resistance will be modelled from glucose, insulin and C-peptide during OGTT. ^25^^,^^26^ Proinsulin will be measured in baseline samples only. Autoantibodies will be measured in participants with OGTT indicative of diabetes to confirm/exclude autoimmune type-1 diabetes.
2. ***HbA1c***: This will be by point-of-care test (Hemocue, Angelholm, Sweden) using fingerprick blood samples for most cohorts to indicate long-term glycaemic control; for the DIVIDS cohort, HbA1c will be measured in venous blood by a commercial lab (Dr. Lal Path Labs, Delhi).
3. ***Abdominal ultrasound*:** Ultrasound scans will be performed by trained sonographers or radiologists in identified health facilities. The participant will be asked to lie on the ultrasound examination bed. They will be asked to breathe in and hold their breath in order to optimise visualization of the pancreas. A curvilinear ultrasound probe (2-5 MHz) will be placed onto the participant’s abdominal wall in the upper abdomen to image the pancreas. Images of the pancreas will be recorded and saved. Parameters obtained from the scans will be pancreas size, duct size, calcification, or other abnormality.
4. ***Exocrine pancreas markers:*** These will be serum lipase in baseline OGTT samples and faecal elastase: Participants will collect faecal samples either in the clinic or bring them from home.
5. ***Urine glucose and ketones by dipstick***: These will be from random urine collection.
6. ***Anthropometry*:** Anthropometry will use standard methods ^27^ conducted by trained staff. At each site technical error of measurement will be assessed and further training provided so that all staff reach acceptable levels of agreement with the local criterion anthropometrist. ^28^ Anthropometric measurements will be weight, height, sitting height, tibia length, circumferences of waist, hip and mid-upper arm, and triceps and subscapular skinfold thicknesses.
7. ***Body composition:*** All sites will use bioelectrical impedance (BIA) for body composition assessment. Lusaka will additionally use air displacement plethysmography (BodPod) and can assess its agreement with BIA.

The following assessments will be conducted in only subsets of consenting participants.

8. ***Detailed OGTT***: The same OGTT protocol will be used as above but, for 80 participants in each of the CICADA and St-ATT cohorts, referred to as the in-depth subset, we will measure additional hormones: glucagon, gastric inhibitory peptide (GIP), glucagon-like peptide-1 (GLP-1) at multiple time points and trypsinogen in baseline samples. The in-depth subsets will be randomly selected from the cohort participants, stratified by whether or not they were recorded as previously malnourished in our databases from earlier follow-ups. In event of refusals, replacements will be from the next in the randomised lists.
9. ***IVGTT:*** This test is the gold standard for estimating the early insulin response. It will be done only in the in-depth subset from CICADA. Results will be compared with OGTT in the same participants in order to better understand the OGTT results. For this test a solution of sterile 50% dextrose, in amounts 0.3 g/kg body weight, will be injected into the antecubital vein over 60 seconds starting at time 0 minutes. Blood samples will be taken at -10, -1, 2, 4, 6, 8, 10 minutes for measurement of glucose, insulin, C-peptide and glucagon.
10. ***CT scans***: The CT studies will be performed in 100 participants from each of the adult cohorts by qualified and experienced radiologists in identified health facilities. Participants will be randomly selected, similarly to the in-depth subsets. Scans will be taken without use of contrast agents. The protocol will assess the following:
  • Pancreatic volume (in cm ^3^)
  • Abnormalities in duct size
  • Presence of cysts or calcification.
    Standardisation of the measurements between sites will be assured by consultation among all site radiologists of 10 scans from each cohort. The studies will be conducted and reviewed for incidental findings at the local site in accordance with the relevant standard operating procedure (SOP).
11. ***Proinsulin-insulin ratio***: This will be done in 120 archived samples from the CICADA cohort which were collected from fasting participants about four years prior to the SAMPA visit.

The DIVIDS child cohort will not have CT scans but 100 children will have MRI scans, funded through a separate project, which will permit examination of pancreas size and structure as well as quantification of visceral fat. Also, in the DIVIDS cohort only, we will measure full blood count, blood lipids and C-reactive protein in baseline samples from the OGTT; this is done for purposes of local ethics and family desire for the information.

### Laboratory analyses

Glucose as part of OGTT and IVGTT tests and HbA1c will be measured using either point-of-care analysers or local labs, depending on the site, in order to provide rapid feedback to participants to enable medical care. Urine ketones will be measured by dipstick in those with suspected diabetes in order to assess the need for short or long-term insulin replacement therapy. Serum lipase, CRP and lipids will be measured at accredited commercial laboratories. Fecal elastase will be measured at each site using stool preparation and ELISA kits from ScheBo (Biotech UK, Lyme Regis, UK).

Other laboratory assays (glucagon, insulin, pro-insulin, C-peptide, GIP, GLP-1 and auto-antibodies) for samples from Mwanza, Lusaka and the Philippines will be conducted together at Rigshospitalet, University of Copenhagen. Since it is not possible to ship samples from India to the UK, Dr. Krogh-Madsen of Rigshospitalet will arrange quality assurance with the Delhi laboratory.

### Data management

Data will be collected using computer-assisted personal interviews. Structured questionnaires programmed in REDCap will be accessed by trained interviewers through tablets. Data will be transmitted to a secure database storage server hosted at the London School of Hygiene and Tropical Medicine (LSHTM). Quality control will be applied at each stage of data handling in accordance with Good Clinical Practice (GCP) requirements to ensure that all data are reliable and have been processed correctly. Access to the data (as uploaded in the database) will be granted to authorised representatives from the sponsor, host institution and the regulatory authorities to permit study-related monitoring, audits and inspections.

### Pre-processing of data and descriptive analyses

Fat and fat-free mass in kg will be divided by height in meters-squared, analogous to determining BMI from weight and height, to give fat mass index (FMI) and fat-free mass index (FFMI).

Socioeconomic status (SES) indices will be derived separately for each site using principal component analysis (PCA) of individual or family assets; the specific assets will differ by site since they have been chosen to be locally appropriate. The first component from the PCA will be divided into terciles for use in analyses.

Site-specific diet patterns will be calculated by PCA of food groups; the number of components to include will be based on eigenvalues and scree plots. ^29^ The level of adherence to individual patterns will be calculated by dividing the components in terciles.

Descriptive analysis will be conducted within cohorts and divided by sex. Means and standard deviations (SDs) will be used to summarise normally distributed data and medians and interquartile ranges (IQR) for non-normally distributed variables. Categorical variables will be described as proportions.

### Statistical analyses and sample size calculations

Analyses related to individual hypotheses are described below. Primary analyses will be conducted for each cohort separately. Pooled analyses using all or some cohorts combined will be conducted based on the individual cohort results, and in order to investigate how age at malnutrition and length of time between malnutrition and outcome assessment influence the associations between malnutrition and pancreas structure and function. Analyses will be guided by the conceptual framework in Figure 1. Sample sizes for the specific hypotheses indicate that, with the available number of participants in the cohorts, we have sufficient power for the main analyses with all participants. For some expensive or labour- and participant-intensive outcomes, we had to consider cost and feasibility and have calculated power for analyses in subsets.

*Hypothesis 1:*
Prior malnutrition is associated with later abnormal pancreatic structure and endocrine and exocrine function.

Participants with and without prior wasting malnutrition, the SAMPA study primary exposure, will be compared within each cohort for all pancreas structure and function outcomes: diabetes by OGTT or HbA1c, exocrine function as indicated by faecal elastase and serum lipase, and pancreas size and presence of abnormalities detected by ultrasound or CT scan. We will initially fit regression models for each outcome with prior malnutrition as the main exposure, controlling for sex and for age as a continuous variable. We will investigate whether there are interactions between prior malnutrition and sex, which is known to be associated with both prior malnutrition and diabetes. We will then investigate whether the associations between malnutrition and the outcomes differ by the following variables:

• Time since malnutrition
• Sitting/standing height ratio as an indicator of early life stunting
• HIV status for African cohorts only (SAM Lusaka, NUSTART Lusaka, CICADA)

Several variables – BMI, FMI, FFMI and diet pattern (using components from PCA as defined above) – have potentially complex relationships with the exposure and outcomes. These variables will be measured at the time of outcome assessment, but are assumed to reflect participants’ nutritional status and diet prior to the outcome. We will start by investigating the associations among diet patterns, BMI, FMI and FFMI at the time of pancreas testing. We will then explore several relationships between these variables, the prior malnutrition exposure, and the outcomes. First, BMI and FMI may be on the causal pathway from wasting malnutrition to diabetes since recovery from wasting malnutrition associated with infection may lead to excess fat deposition, ^30^^,^^31^ and since people previously severely malnourished may, if it becomes possible later, choose to gain weight as a buffer against future food shortages. Second, BMI, FMI and diet patterns could be modifiers of the association between prior malnutrition and diabetes, as seen previously for diet pattern and BMI in Chinese famine survivors. ^32^ Third, exocrine pancreas abnormalities which result in fat malabsorption or intestinal discomfort may induce participants to modify their diets which could result in changes to BMI, FMI or FFMI.

We will conduct exploratory analyses to investigate the mechanisms through which the exposure variable, malnutrition, is associated with pancreas outcome variables. For example, decreased production of incretins by gut epithelial cells may be associated with both exocrine pancreas function, which could affect rate of absorption of nutrients which trigger incretin secretion, and insulin secretion.

***Sample size***: Large variations in prevalence of impaired glucose tolerance (OGTT≥7.8 mmol/L *)* or high fasting glucose in never malnourished (2%-11%) make estimates imprecise. The absolute difference in prevalence of hyperglycaemia in those with prior malnutrition versus those without was 3% to 9% in these studies. ^32^^–^^34^ Assuming an intermediate prevalence of OGTT ≥7.8 mmol/L of 7% in those without malnutrition, the CICADA and St-ATT cohorts individually have 80% power to detect a difference in prevalence of 7.5% in those with malnutrition. For continuous variables, *i.e.* glucose, hormones, faecal elastase and serum lipase, the available sample size in the smallest cohort will be sufficient to determine differences of 0.4 times the standard deviation with 90% power. Expected differences in pancreas volume were calculated using volume normalised to kg body weight to account for the participant age and size range. ^35^ A total of n=100 per cohort (assuming a standard deviation of 0.3 mL/kg) will provide 90% power to detect differences of 0.2 mL/kg between those with/without prior malnutrition, which is a smaller difference than that expected between people with and without diabetes. ^35^^,^^36^

*Hypothesis 2:*
Prior malnutrition is associated with pancreatic calcification which in turn is associated with diabetes.

To investigate whether prior malnutrition is associated with pancreas calcification, from each adult cohort we will randomly select for CT scans 50 participants who were exposed to malnutrition and 50 controls who were not exposed. The presence of calcification or other pancreas abnormalities on CT will be compared between those with or without prior malnutrition.

In order to increase sample size for this and for the second part of the hypothesis comparing calcification with diabetes, results from CT scans in the subset of participants with these will be compared with the ultrasound results from the same participants to indicate whether abnormalities determined by ultrasound, e.g. pancreas body or duct shape, correspond to calcification by CT scan. We will then compare the proportion of abnormalities based on ultrasound or CT scan among those with and without diabetes.

***Sample size***: Population prevalence estimates for pancreatic calcification appear unavailable so we have not performed any sample size calculations. Pancreatic calcifications are seen in fibro-calculous pancreatic diabetes ^7^ but these studies have been conducted on participants selected for their clinical presentation.

*Hypothesis 3:*
The abnormal glucose regulation seen after malnutrition is associated with relative insulin deficiency with or without insulin resistance.

A deeper understanding of associations between prior malnutrition and the underlying glucose metabolic pathways will be explored through modelling biochemical measures over the course of the two-hour OGTT. Glucose, insulin and C-peptide data from OGTT will be used to model pancreatic cell functionality according to published methods in order to determine a set of indices quantifying: first phase, second phase, and total insulin secretion, ^37^ whole-body and hepatic insulin clearance, ^38^ and insulin sensitivity. ^25^^,^^39^ For the child cohorts for whom only three blood samples are available over the two-hour OGTT, we will use methods designed to determine insulin sensitivity from sparsely sampled OGTTs. ^26^ The IVGTT data from the CICADA cohort subset can be similarly modelled to estimate insulin sensitivity and insulin secretion. Comparison of the indices estimated from the two tests will provide information as to whether altered intestinal function, *e.g.* glucose absorption or incretin secretion, influences insulin secretion or sensitivity in participants previously malnourished or not.

***Sample size***: We will use individual data from all SAMPA adult participants and, for children who have only three sample points during OGTT, we will combine data from groups of participants. ^26^ Most previous studies in this area include 10-100 people per group, whereas we will be able to study all SAMPA participants.

*Hypothesis 4:*
An abnormal fasting pro-insulin/insulin ratio is associated with later development of diabetes in adults who had malnutrition and infection.

***Analysis***: We will use the detailed data and samples available from CICADA to investigate whether the proinsulin/insulin ratio in CICADA samples collected from fasted participants about four years prior to the SAMPA visit is associated with diabetes or prediabetes at the SAMPA visit. This will be a nested case-control study where cases are 60 CICADA participants with current diabetes by OGTT and controls are 60 CICADA participants with normal OGTT. These participants will be selected by stratified random sampling. Logistic regression will be used, controlling for age and sex, to determine if prior proinsulin/insulin ratio is associated with later diabetes.

***Sample size***: The sample size was a function of available cases and consumable costings. A sample of 120 (1:1 ratio of cases and controls) will provide 90% power to detect a difference of 0.04 in the fasting proinsulin/insulin ratio (assuming an SD of 0.07) which is smaller than that found between people with and without type 2 diabetes. ^40^

### Dissemination plans for study outcome

Lead investigators from each of the study sites will disseminate the study findings to the participants and their families, to local clinicians working in nutrition, diabetes or related fields, and to the relevant government departments. Details of this dissemination will vary by study site and will be done according to sites’ usual protocols. Academics will learn about the research methods and findings through open-access, peer-reviewed journal articles, conference presentations, both international and in the participant countries, and seminar series and other platforms for exchanging information with staff and students within the partner institutions. They will then be able to take the results in various directions which will have impact beyond the study team’s expertise.

Data can be made available on request from the Principal Investigator for bona fide research conducted in accordance with protocols and stipulations in the project’s ethical approvals.

### Ethical considerations and regulatory approval

The study will be conducted in compliance with the Declaration of Helsinki's principles, as well as all applicable regulations and the 1996 ICH Guidelines for Good Clinical Practice (CPMP/ICH/135/95). The protocol, informed written consent and assent forms, participant information sheet and other required materials have been approved by the appropriate Research Ethics Committees ( Table 2). If required, the investigators will obtain approval from these committees for all substantial amendments to the original approved documents. Permission has already been obtained from previous cohort follow-ups to use data and samples for subsequent research and has been agreed for SAMPA as well.

**Table 2.** Research Ethics Committees.

Country	Ethics boards	Ethics approval number
UK	London School of Hygiene and Tropical Medicine Ethics committee	22792-2
Tanzania	Medical Research Coordinating Committee at the National Institute for Medical Research	NIMR/HQ/R.8a/Vol.IX/3686
Zambia	University of Zambia Biomedical Research Ethics Committee	1686-2021
India	Ethics Committee at the Institute of Home Economics	IHE/2021-22/Admin/Ethics.com/684(A)
Philippines	St. Frances Cabrini Medical Center - Asian Eye Institute (SCMC-AEI Ethics Review Committee)	2021-012

Children will have only three-sample OGTT and neither they nor pregnant women will be offered CT scans. All participants found during clinic visits or on later review of ultrasound and CT scans to have diabetes or other illnesses requiring treatment will be referred to local clinical facilities and, if required, offered a consultation with an appropriate specialist.

### Policy implications

If low insulin production, either with or without insulin resistance, is an important cause of diabetes in populations in which prior wasting malnutrition is common, then diagnosis and treatment regimes may require modification. By providing insights into hormonal changes during an OGTT, the project will provide information as to how diagnostic protocols could be modified, e.g. by looking at other time points than 120 minutes in an OGTT or including measurement of insulin or C-peptide. The usual current first line treatment in many settings is metformin but modified treatment protocols could include drugs promoting insulin secretion with earlier progression to injected insulin therapy.

## Conclusions

There is some evidence that wasting malnutrition in children and adults may leave lasting pancreatic damage and increase the risk of diabetes later in life, although little is known about the mechanisms involved. SAMPA will lead to better understanding of the long-term impacts of malnutrition as well as the manifestation and underlying metabolism of diabetes in Africa and Asia. Even if no significant link between wasting malnutrition and later diabetes is established, the research will improve understanding of both the long-term consequences of malnutrition and the phenotype of glucose dysregulation in Africa and Asia. It will thus result in better health care for both malnourished individuals and people with diabetes.

## Data availability

No data are associated with this article.
