# Supplementary material for: Long-term health after Severe Acute Malnutrition in children and adults- the role of the Pancreas (SAMPA): Protocol
Source: F1000Res. 2022 Oct 3;11:777. Originally published 2022 Jul 12. [Version 2] doi: 10.12688/f1000research.123389.2 (PMC9577280; doi:10.12688/f1000research.123389.2)
Supplement: Peer review history

# Peer review: Reviewer response for version 2

**DOI:** 10.5256/f1000research.139279.r152297

**Published:** 2022-10-17

**Authors:** Ishu Kataria

**Affiliations:** 1 Center for Global Noncommunicable Diseases, RTI International, New Delhi, Delhi, India

No additional comments. I am satisfied with the authors' responses.

Is the study design appropriate for the research question?

Yes

Is the rationale for, and objectives of, the study clearly described?

Yes

Are sufficient details of the methods provided to allow replication by others?

Partly

Are the datasets clearly presented in a useable and accessible format?

Not applicable

Reviewer Expertise:

Noncommunicable Diseases; NCD policy

I confirm that I have read this submission and believe that I have an appropriate level of expertise to confirm that it is of an acceptable scientific standard.

# Peer review: Reviewer response for version 2

**DOI:** 10.5256/f1000research.139279.r152296

**Published:** 2022-10-06

**Authors:** David J. Hill

**Affiliations:** 1 Lawson Health Research Institute, St Joseph Health Care, London, ON, Canada; 2 Western University, Ontario, Canada

The article has been much improved by adding explanations as to why many of the measurements will be performed. I have no further comments.

Is the study design appropriate for the research question?

Yes

Is the rationale for, and objectives of, the study clearly described?

Yes

Are sufficient details of the methods provided to allow replication by others?

Partly

Are the datasets clearly presented in a useable and accessible format?

Yes

Reviewer Expertise:

Diabetes, pancreatic islet development and function. fetal programming of adult metabolic disease, diabetes in pregnancy, beta cell biology

I confirm that I have read this submission and believe that I have an appropriate level of expertise to confirm that it is of an acceptable scientific standard.

# Peer review: Reviewer response for version 1

**DOI:** 10.5256/f1000research.135491.r149409

**Published:** 2022-09-16

**Authors:** Ishu Kataria

**Affiliations:** 1 Center for Global Noncommunicable Diseases, RTI International, New Delhi, Delhi, India

This study will assess the association of prior wasting malnutrition on endocrine and exocrine pancreatic structure and function. It will include 3700 participants from existing observational cohorts in four LMICs.  

 Based on reviewing the protocol manuscript, here are some suggestions that would strengthen the paper; and overall study:

1. With regards to participant recruitment, there are two cohorts out of six, where the sample will include adolescents. Although it is mentioned that parental consent will be obtained, an assent is recommended in case of adolescents. 
2. For the OGTT analysis, how will the sub-groups be selected? 
3. The investigators mention in their dissemination strategy for the project that they will be sharing the findings with participants and their families as well as with the relevant government departments. While there is clarity on the methods identified to reach academics and local clinicians, it is pertinent that there is a plan for dissemination for the abovementioned groups as the methods would be completely different. 
4. It will be important to consider inclusion of patient perspectives in the research. Patients have multiple ongoing interactions with the health system over long periods of time and can provide useful inputs in terms of  patient acceptability of aspects of study design, and also review draft patient information materials.
5. The study aims to generate evidence for future policies and programmes for malnutrition and diabetes; however, description about how that linkage will happen is missing. Suggest adding a paragraph on policy implications of research in the protocol paper.

Is the study design appropriate for the research question?

Yes

Is the rationale for, and objectives of, the study clearly described?

Yes

Are sufficient details of the methods provided to allow replication by others?

Partly

Are the datasets clearly presented in a useable and accessible format?

Not applicable

Reviewer Expertise:

Noncommunicable Diseases; NCD policy

I confirm that I have read this submission and believe that I have an appropriate level of expertise to confirm that it is of an acceptable scientific standard, however I have significant reservations, as outlined above.

# Response

**Published:** 2022-09-29

**Authors:** Sana Ahmed

We appreciate you taking the time to read over and thoughtfully comment on our work. We are really grateful for your contribution. Below are the detailed answers (in bold) to your remarks:

- With regards to participant recruitment, there are two cohorts out of six, where the sample will include adolescents. Although it is mentioned that parental consent will be obtained, an assent is recommended in case of adolescents. 

**As we have mentioned under ‘ethical considerations and regulatory approvals’ we would be getting assent from the adolescent participants in addition to the parental consent. **

- For the OGTT analysis, how will the sub-groups be selected? 

**All participants will have OGTT undertaken, the main difference being that there will be only 3 blood sampling time points for children, 7 for adults. For the in-depth sub-samples in some of the adult cohorts, we will randomly select these, stratified by whether or not they were previously malnourished, as recorded in our databases from earlier cohort follow-ups. This information, which also applies to the subsets for CT scans, has been added to the description of tests performed only on subsets of participants. **

- The investigators mention in their dissemination strategy for the project that they will be sharing the findings with participants and their families as well as with the relevant government departments. While there is clarity on the methods identified to reach academics and local clinicians, it is pertinent that there is a plan for dissemination for the abovementioned groups as the methods would be completely different. 

**All the reports from the several investigations that will be conducted will be duly shared and discussed with the participants, their families, local clinicians, and health care managers. The format for this dissemination will vary by study site and will be done according to usual practice at the sites. This information has been added to that section of the text. **

- It will be important to consider inclusion of patient perspectives in the research. Patients have multiple ongoing interactions with the health system over long periods of time and can provide useful inputs in terms of patient acceptability of aspects of study design, and also review draft patient information materials.

**While formal patient input into research is becoming common in some places, it is not currently the norm at any of the study sites. We are developing such approaches and setting up Community Advisory Boards in several sites; however, this strategy was not in place when SAMPA was being designed. On the other hand, SAMPA participants are all from cohorts with whom the researchers at each site have had long contact. Participants are thus comfortable talking to the staff and the staff thus gains insight into their perspectives. We often use such information gathered to generate or modify research plans.  **

- The study aims to generate evidence for future policies and programmes for malnutrition and diabetes; however, description about how that linkage will happen is missing. Suggest adding a paragraph on policy implications of research in the protocol paper.

**We have added the following paragraph on policy implications near the end of the manuscript:**

**If low insulin production, either with or without insulin resistance, is an important cause of diabetes in populations in which prior wasting malnutrition is common, then diagnosis and treatment regimens may require modification. By providing insights into hormonal changes during an OGTT, the project will provide information as to how diagnostic protocols could be modified, e.g. by looking at other time points than 120 minutes in an OGTT or including measurement of insulin or C-peptide. The usual current first-line treatment in many settings is metformin but modified treatment protocols could include drugs promoting insulin secretion with earlier progression to injected insulin therapy.**

# Peer review: Reviewer response for version 1

**DOI:** 10.5256/f1000research.135491.r147874

**Published:** 2022-08-19

**Authors:** David J. Hill

**Affiliations:** 1 Lawson Health Research Institute, St Joseph Health Care, London, ON, Canada; 2 Western University, Ontario, Canada

The hypothesis is that wasting malnutrition either in childhood or adulthood will cause long-term impacts on exocrine and endocrine pancreatic function following nutritional recovery, and that this will result in an increased risk of diabetes. This study will examine almost 4,000 individuals who experienced serious malnutrition from a number of existing study cohorts recruited in Zambia, Tanzania, Philippines, and India under the SAMPA International Program. Pancreas size, structure, and degree of calcification will be assessed by ultrasound and CT scan; exocrine function will be assessed by fecal elastase and serum lipase measurements and endocrine function by HbA1c measurement, blood glucose, insulin and C-peptide concentrations during an OGTT. Sub-sets of patients will be selected for analysis of insulin, pro-insulin, glucagon and incretin excursions during GTTs. Pancreatic size and function outcomes will be compared between subjects with and without prior wasting malnutrition. The impact of sex, age, time since malnutrition, current BMI and diet will be analyzed.

 This is a very ambitious study covering a number of existing longitudinal cohorts. A major strength is the real-world approach to health outcomes in populations that experience serious famine, which is important for the planning of future health provision. The sample size is impressive but needs to be given the number of confounders that will exist. I may have missed it but will subjects who have already developed clinical diabetes be excluded?

 With respect to pancreatic function I think better justification needs to be provided as to why ultrasound and CT estimation of anatomy and ductal structure need to be undertaken. Is there evidence that total pancreas volume is associated with either endocrine or exocrine sufficiency. Certainly pancreas size does not give any indication of endocrine cell mass since the islets represent less than 5% of pancreas cell mass. It is stated that CT will allow an estimate of duct size, but what does this imply? The degree of calcification will be measurable but it is not explained why this might cause an alteration in either exocrine or endocrine function. What might be useful if possible is to use contrast micro-CT to measure changes in the vascular density of the pancreas.

 The timing and duration of malnutrition relative to age is likely to be very important with respect to the risk of adult diabetes and should be given more attention. Beta cell mass in the pancreas increases until adolescence through the potential for beta cell proliferation although this declines with age. After adolescence gain of beta cell mass is minimal. In terms of plasticity of beta cell mass this can recover during childhood from a metabolic insult such as malnutrition but this is likely to be minimal after adolescence. This, documentation of the age of onset of the malnutrition, duration and intensity, and recovery will be crucial to the metabolic phenotype and risk of adult diabetes. As four of the six cohorts are already adults it should be made clear what degree of precision around the historical documentation of these measurements will be available.

 While the detailed analysis from OGTT and i.p.GTT will be very informative in the sub-group analysis it is not clear how the sub-groups will be selected. Will they sample from the subjects found to have the most abnormal pancreas parameters, or from a group that represents median measurements? It is stated that both glucagon and insulin/proinsulin excursions will be measured during GTTs but the biological relevance of these measurements is not given. Is the proinsulin/insulin ratio meant to be a measure of beta cell stress? If so then there should also be changes in the ratio of first to second phase insulin release as the stressed pancreas will likely have a smaller pool of readily releasable insulin granules. Is the glucagon measurement meant to indicate a hyperglucagonemia as often found in T2D?

 These comments are made in the constructive spirit of getting the most out of this very important study and how it is portrayed in the manuscript.

Is the study design appropriate for the research question?

Yes

Is the rationale for, and objectives of, the study clearly described?

Yes

Are sufficient details of the methods provided to allow replication by others?

Partly

Are the datasets clearly presented in a useable and accessible format?

Yes

Reviewer Expertise:

Diabetes, pancreatic islet development and function. fetal programming of adult metabolic disease, diabetes in pregnancy, beta cell biology

I confirm that I have read this submission and believe that I have an appropriate level of expertise to confirm that it is of an acceptable scientific standard, however I have significant reservations, as outlined above.

# Response

**Published:** 2022-09-29

**Authors:** Sana Ahmed

Thank you for your thorough and insightful review of our manuscript. We sincerely appreciate your invested time and efforts. Please find below our detailed responses (in bold) to your comments:

- The sample size is impressive but needs to be given the number of confounders that will exist. I may have missed it but will subjects who have already developed clinical diabetes be excluded?

**We have not excluded those with diabetes. However, very few participants were known in advance to have diabetes since in only two of the six cohorts, CICADA and St-ATT, had there been previous studies of diabetes. Furthermore, for St-ATT participants, diabetes screening was undertaken when participants were acutely ill with tuberculosis which may have impacted on the diagnosis. For all cohorts, we are collecting data on whether participants have been previously diagnosed with diabetes and whether they are on any diabetes medication.**

  

- With respect to pancreatic function I think better justification needs to be provided as to why ultrasound and CT estimation of anatomy and ductal structure need to be undertaken. Is there evidence that total pancreas volume is associated with either endocrine or exocrine sufficiency. Certainly pancreas size does not give any indication of endocrine cell mass since the islets represent less than 5% of pancreas cell mass. It is stated that CT will allow an estimate of duct size, but what does this imply? The degree of calcification will be measurable but it is not explained why this might cause an alteration in either exocrine or endocrine function. What might be useful if possible is to use contrast micro-CT to measure changes in the vascular density of the pancreas.

**There is no doubt that pancreas size is considerably reduced in type 1 diabetes despite this being a beta-cell targeted autoimmune disease, with islets constituting less than 5% of the overall pancreas volume. There is also accruing evidence for reduced pancreatic volume in type 2 diabetes; see, for example, Al-Mrabeh et al. Lancet Diabetes Endocrinology 2020; 8(12): 939-948, or Virostko et al., Diabetes Care 2019; 42(2): 248-257. We hypothesise that smaller pancreas size may reflect reduced islet mass - due to reduced growth during development in those with malnutrition occurring under the age of 20 years or due to loss of previously acquired mass. Pancreatic calcification is associated with a 3-fold increased risk of diabetes in chronic pancreatitis and ductal structure will be examined to further characterise the potential underlying pancreatic disease. Our ethical approvals at the sites do not permit contrast CT scans. Our purpose here is to define, we believe for the first time, whether, and what sort of, changes develop in the pancreas attributable to prior malnutrition. We, therefore, have to try and capture all possible changes which may occur.**

  

- The timing and duration of malnutrition relative to age is likely to be very important with respect to the risk of adult diabetes and should be given more attention. Beta cell mass in the pancreas increases until adolescence through the potential for beta cell proliferation although this declines with age. After adolescence gain of beta cell mass is minimal. In terms of plasticity of beta cell mass this can recover during childhood from a metabolic insult such as malnutrition but this is likely to be minimal after adolescence. This, documentation of the age of onset of the malnutrition, duration and intensity, and recovery will be crucial to the metabolic phenotype and risk of adult diabetes. As four of the six cohorts are already adults it should be made clear what degree of precision around the historical documentation of these measurements will be available.

**We agree completely that the potential for beta cell proliferation declines with age, but hypothesise that malnutrition may have a greater effect on beta cell mass in those affected during growth and development due to reduced final beta-cell mass persisting into adulthood. The data on malnutrition were collected prospectively for 5 of the 6 cohorts and thus exact ages and degrees of malnutrition are known. The cohorts were either recruited based on being malnourished (DIVIDS, NUSTART Lusaka, St-ATT) or were recruited for longitudinal studies (CICADA, CLHNS) so we have nutritional status documented over several years in the past. For SAM Lusaka we recruited from hospital records when the children were admitted for severe acute malnutrition several years before our first contact with the children.**

  

- While the detailed analysis from OGTT and i.p.GTT will be very informative in the sub-group analysis it is not clear how the sub-groups will be selected. Will they sample from the subjects found to have the most abnormal pancreas parameters, or from a group that represents median measurements? It is stated that both glucagon and insulin/proinsulin excursions will be measured during GTTs but the biological relevance of these measurements is not given. Is the proinsulin/insulin ratio meant to be a measure of beta cell stress? If so then there should also be changes in the ratio of first to second phase insulin release as the stressed pancreas will likely have a smaller pool of readily releasable insulin granules. Is the glucagon measurement meant to indicate a hyperglucagonemia as often found in T2D?

**In order to test our primary hypothesis that prior malnutrition has long-term effects on pancreas function, we will randomly select, stratified by prior malnutrition or not, from the full list of recruits to the relevant cohorts. This information, for CT scans as well, has been added to the section on tests performed in subsets of participants.**

**Proinsulin will be measured in fasted samples just before the OGTT and its ratio with insulin calculated; this will indeed be used as a measure of** **beta** **-cell stress or potentially phenotypic shift induced by the beta-cell-specific impact of malnutrition. Our models of the full glucose, insulin and C-peptide data from all OGTT time points will provide further details of early and later phase insulin release. Several of the authors are building the models from their clinical studies in cystic fibrosis, a model disease likely to have a similar pancreatic phenotype as we look for in SAMPA. We recognise that the underlying pathophysiology of cystic fibrosis is completely different; cystic fibrosis is, however, also a disease of malnutrition combined with exocrine dysfunction. We suggest the proinsulin-to-insulin ratio is a good general indicator of stressed beta cells, as previous studies reported increased proinsulin-to-insulin ratios in both persons with and without cystic fibrosis with insufficient insulin secretion. We hypothesize that dysfunctional beta cells play an important role in the progression of abnormal glucose tolerance towards diabetes.**

**Yes, the glucagon measurement is to detect whether an oral intake of glucose results in inappropriately high plasma concentrations of glucagon as in T2D. It is as yet unknown whether the same aberrant response will be seen in patients with prior malnutrition or current pancreas dysfunction.**

**We have added text to the section on outcome measures to clarify our reasons for choosing these.**
